# The Cardiohepatic Axis in Cirrhosis

**DOI:** 10.1016/j.jacbts.2025.101314

**Published:** 2025-07-28

**Authors:** Saad A. Ali, Kyle Frick

**Affiliations:** Division of Cardiovascular Medicine, Department of Medicine, Indiana University School of Medicine, Indianapolis, Indiana, USA

**Keywords:** advanced cardiac imaging, cardiohepatic axis, cirrhotic cardiomyopathy, coronary artery disease, end stage liver disease, orthotopic liver transplantation

## Abstract

Cardiac dysfunction in the setting of cirrhosis is often overlooked despite its impact on contractility, diastolic dysfunction, and electrophysiological abnormalities. This review examines the complex interactions of the cardiohepatic axis, with a particular focus on how liver disease impacts heart function. We highlight the roles of portal hypertension, systemic inflammation, hemodynamic changes, and neurohormonal signaling to shed light on this important connection. The underlying mechanisms for impaired cardiac performance involve both molecular and structural changes. Diagnosis remains challenging because it is frequently subclinical; however, advanced imaging, biomarkers, and electrocardiographic changes have improved early detection. Clinically, this syndrome has significant implications, particularly in decompensated cirrhosis and periods of increased myocardial stress. Although no standardized treatment guidelines exist, orthotopic liver transplantation remains the most effective intervention and often results in cardiovascular recovery. A multidisciplinary approach between cardiologists, hepatologists, and transplant surgeons is crucial to improving patient outcomes and optimizing long-term management.

## Highlights

• The cardiohepatic axis links heart and liver dysfunction, with cirrhosis causing under-recognized and latent cardiac dysfunction.
• This review examines cirrhosis-induced cardiac dysfunction, emphasizing diagnostic challenges and the need for rigorous preoperative cardiovascular assessment.
• Advanced imaging and biomarkers aid early detection, but standardized treatments are lacking; liver transplantation remains the definitive intervention.
• Future research should clarify molecular mechanisms, refine diagnostics, and develop targeted therapies to improve patient outcomes.

The cardiohepatic axis describes the synergistic relationship between the heart and liver, highlighting the physiological, pathological, and biochemical communication that allows these organs to influence each other’s function. It is well known that heart failure can precipitate congestive hepatopathy or ischemic hepatitis caused by passive venous congestion or impaired cardiac output. Conversely, liver disease can lead to cardiac abnormalities that often go unrecognized, including impaired contractility under stress, diastolic dysfunction, and electrophysiological disturbances that occur in the absence of existing cardiac disease (Central Illustration). Collectively, cardiac dysfunction in end-stage liver disease (ESLD) is an entity that has been termed cirrhotic cardiomyopathy.

**Central Illustration:**
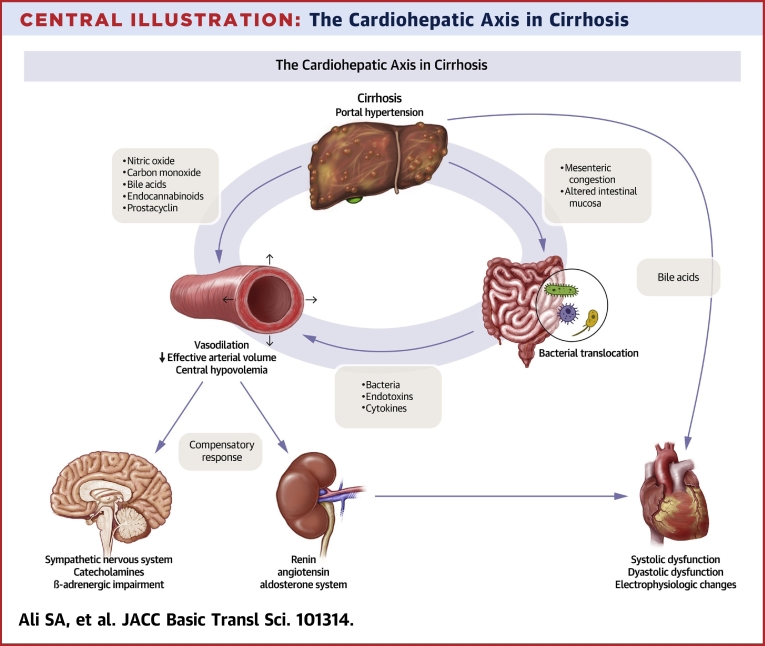
The Cardiohepatic Axis in Cirrhosis RAAS = renin angiotensin aldosterone system.

This review provides a comprehensive overview of the complex connections between cardiac and hepatic health. It will explore the epidemiology and pathophysiology of these interactions, in addition to detailing the various forms of cardiac dysfunction observed in liver disease. Finally, this review will examine the diagnostic challenges, clinical implications, and treatment approaches of this complex clinical syndrome.

## Epidemiology

Cardiovascular disease in the setting of ESLD attracts significant attention because of its association with poor outcomes, especially in patients undergoing orthotopic liver transplantation (OLT). Cardiac dysfunction is often subclinical and underdiagnosed, which increases the risk for progressing toward hemodynamic instability, heart failure, and mortality when exposed to stress. In fact, overt heart failure accounts for 7% to 24% of early post-OLT deaths.^1^ Similarly, cardiac decompensation is the leading cause of mortality following transjugular intrahepatic portosystemic shunt in patients with cirrhosis, with 20% being hospitalized for heart failure within the first year following the procedure.^1^ These findings highlight the importance of thoroughly assessing cardiac function in patients with ESLD.

## Pathophysiology

### Cirrhosis-associated hemodynamic changes

Cirrhosis leads to significant structural and functional alterations in the liver that result from fibrogenesis, inflammation, angiogenesis, and the progressive loss of parenchymal cells.^2^ These changes lead to increased hepatic vascular resistance, which plays a central role in the development of portal hypertension. The combined effects of portal hypertension and the systemic inflammatory state of cirrhosis form the foundation for many of the well-established complications in cirrhotic patients, including their significant contribution to cardiac dysfunction.

Portal hypertension arises from 2 primary mechanisms: increased resistance to portal venous inflow from structural and functional changes within the intrahepatic circulation, and increased portal venous flow, caused by splanchnic vasodilation and an increase in cardiac output. Regarding increased hepatic vascular resistance, approximately 70% of the resistance arises from scarring caused by the accumulation of excessive extracellular matrix from the effects of activated hepatic stellate cells that replace functional liver tissue. The remaining 30% of the resistance comes from vasoconstriction mediated by hepatic stellate cells and vascular smooth muscle cells in response to elevated levels of vasoconstrictors like angiotensin II and endothelin-1. These changes are compounded by endothelial dysfunction that results in reduced nitric oxide production in sinusoidal endothelial cells.^3^ Together, these processes lead to a gradual rise in intrahepatic vascular resistance, resulting in portal hypertension and the development of portal-systemic shunts.

Whereas intrahepatic vasculature encounters increased vascular resistance, splanchnic circulation, on the other hand is characterized by vasodilation from molecules such as nitric oxide, endocannabinoids, and carbon monoxide.^4^ Specifically, cirrhotic patients exhibit chronically elevated nitric oxide levels, and this molecule has been recognized as the primary driver for vasodilation in both the splanchnic and systemic circulation. This extrahepatic vasodilation increases portal vein flow, which worsens portal hypertension. Ultimately, this leads to mesenteric congestion that results in altered intestinal mucosal permeability and translocation of bacteria, endotoxins, and cytokines into the bloodstream.^5^ This vasodilatory state also contributes to central hypovolemia and reduced effective arterial circulating volume. To maintain circulatory function, compensatory neurohumoral mechanisms are triggered, including activation of the renin-angiotensin-aldosterone system, sympathetic nervous system, and catecholamine release. These responses increase heart rate and cardiac output, ultimately driving the hyperdynamic circulation characteristic of ESLD.^4^

### Cirrhosis associated cardiovascular changes

Cardiac dysfunction in cirrhosis is characterized by several key features that include impaired systolic and diastolic function, left ventricular (LV) hypertrophy, and a blunted compensatory response to stress despite an increased resting cardiac output. Furthermore, electrophysiological abnormalities, such as prolonged QT intervals and electromechanical uncoupling, are commonly observed.^6^

#### Systolic dysfunction

Systolic dysfunction in patients with cirrhosis is linked to increased morbidity, mortality, and complications of decompensated cirrhosis such as hepatorenal syndrome.^7^ At rest, patients with cirrhosis often display normal or hyperdynamic systolic function due the compensatory responses outlined in the previous text. However, during periods of stress, such as physical, infectious, or surgical, studies have demonstrated the failure of cardiac compensatory mechanisms in responding to this hyperdynamic state. For example, a study found reduced cardiovascular performance with impaired inotropic and chronotropic responses to exercise in cirrhotic patients.^8^ Another study showed impaired ventricular function in patients with alcoholic cirrhosis when exposed to pharmacologically induced stress.^9^ The immediate post-OLT state consists of significant cardiovascular stress from circulatory shifts that result in abrupt changes in preload and afterload. Studies have reported a reduction in LV ejection fraction post-OLT because of a sudden rise in afterload and reversal of hyperdynamic circulation.^10^

The inability to appropriately sustain contractility under these conditions can lead to the progression from subclinical dysfunction to overt heart failure. This may be explained by the fact that the heart is operating at its maximum preload and contractile reserve at rest, restricting its ability to further increase cardiac output under stress. Several molecular mechanisms contribute to impaired LV contractility, including reduced β-adrenergic signaling, altered ion channel function, and increased levels of endogenous cannabinoids and other cardio-depressant factors.

The β-adrenergic receptor system is widely recognized for the role it plays in regulating contractility. Catecholamine stimulation of β-adrenoceptors triggers a cascade of events that activate the cyclic AMP second-messenger pathway. This leads to phosphorylation of key proteins that ultimately mediate inotropic, chronotropic, and dromotropic effects.^11^ The circulatory shifts seen in cirrhosis trigger repeated activation of the sympathetic nervous system and subsequent catecholamine release to preserve circulatory function. Over time, this results in the down-regulation and desensitization of β-adrenergic receptors.^12^ In fact, cirrhotic rats have been shown to have a significantly lower density of β-adrenoceptors and require higher doses of isoprenaline to increase heart rate. This suggests β-adrenoceptors down-regulation reduces myocardial responsiveness to catecholamines.^13^

Elevated levels of endocannabinoids and other vasoactive mediators contribute to worsening LV dysfunction in cirrhotic patients. The up-regulation of the cannabinoid signaling pathway has been implicated in the pathophysiology of hypotension and negative inotropy associated with cirrhosis. The endotoxemia commonly seen in these patients activates vascular CB1 receptors, which further exacerbates hypotension. Moreover, endocannabinoids exert a direct negative inotropic effect by interacting with CB1 and CB2 receptors, which are inhibitory G-protein-coupled receptors. This interaction inhibits adenylate cyclase activity, leading to reduced cAMP levels and diminished calcium influx into cardiomyocytes, causing impaired cardiac contractility.^14^ In addition to the effects of endocannabinoids, elevated serum bile acid concentrations decrease fatty acid oxidation in cardiomyocytes and have been shown to impair chronotropy and inotropy.^15^

#### Diastolic dysfunction

LV diastolic dysfunction is characterized by impaired LV relaxation and reduced compliance, which affects LV filling and results in elevated intracardiac pressures. In patients with cirrhosis, LV diastolic dysfunction often precedes systolic dysfunction and is influenced by overstimulated neurohormonal mechanisms, hyperdynamic circulation, and the inflammatory state seen in cirrhosis.^4^ Over time, histologic changes occur, including myocardial fibrosis, interstitial edema, LV hypertrophy, and nuclear and cytoplasmic vacuolation of cardiac myocytes.^4^

Although the underlying mechanisms for LV diastolic dysfunction in cirrhotic patients remain unclear, some studies have suggested that abnormalities in sarcomeric titin may play a role in this process. Titin is an exceptionally large protein within muscle cells, stretching from the Z disk to the M-line of the sarcomere, the fundamental unit of muscle contraction.^16^ Titin plays an important role in the elastic characteristics of relaxed striated muscle. As a primary source of passive tension, it significantly impacts the diastolic stiffness of cardiomyocytes.^17^ In cardiac muscle, there are 2 distinct titin isoforms, N2BA and N2B, that differ in size and mechanical properties. Studies on titin isoform ratios in cardiac disease have shown varying results. One study found that up-regulation of N2BA:N2B ratio in end-stage heart failure was associated with reduced diastolic stiffness.^16^ On the other hand, another study found no difference in N2BA:N2B expression between control subjects and cirrhotic hearts. However, the study did observe a reduction in titin phosphorylation in cirrhotic hearts, leading to increased passive tension in the LV.^17^

In addition to intracardiomyocyte contributions to diastolic impairment, extrinsic etiologies have also been identified. Increases in extracellular matrix components and more specifically, fibrillar collagen, have been linked to the fibrotic changes of the myocardium that contribute to LV stiffness and wall thickness.^18^ Patients with heart failure with preserved ejection fraction have increased collagen type 1 expression and enhanced collagen cross linking, both of which are associated with impaired diastolic function.^19^ In cirrhotic rat models, the more stiff collagen isoform, collagen type I, was found to be more predominant compared with the more compliant collagen type III isoform.^17^

#### Electrophysiologic disturbances

QT interval prolongation is one of most commonly observed electrophysiologic abnormalities in patients with cirrhosis with an estimated prevalence that exceeds 40% regardless of the underlying etiology.^20^ Prolongation of the QT interval is clinically significant because it carries an increased risk of ventricular arrhythmia and sudden cardiac death. Moreover, this finding has been shown to correlate with the severity of cirrhosis, highlighting its prognostic importance.^20^ Several mechanisms for the development of QT prolongation in cirrhosis have been proposed. Altered cardiomyocyte plasma membrane fluidity, often seen in cirrhosis, can impair the function of membrane-bound ion channels. Supporting this, a study showed decreased expression of 2 types of K^+^ channels in ventricular myocytes from cirrhotic rats, providing a mechanistic basis for QT prolongation in this setting.^21^ In light of this finding, avoidance of QT prolonging medications is recommended.

Similarly, alterations in Ca^2+^ homeostasis caused by changes in membrane fluidity can lead to a mismatch between electrical and mechanical systole, a condition referred to as electromechanical uncoupling. In cirrhotic rats, a reduction in excitation-contraction coupling was attributed to decreased density of voltage-gated L-type Ca^2+^ channel expression on cardiomyocytes.^22^ Furthermore, these models have shown an extended electromechanical delay and increased ratio of the pre-ejection period to LV ejection time. Studies have shown that following exercise, cirrhotic patients experience blunted chronotropy and diastolic blood pressure.^23^ This finding highlights potential mechanisms of the impaired cardiovascular response to stress seen in cirrhotic patients.

Chronotropic incompetence is defined as the failure to achieve at least 85% of the age-predicted maximal heart rate during exercise or increased metabolic demand and is another electrophysiologic abnormality seen in patients with cirrhosis.^24^ This condition is thought to arise from autonomic dysfunction, caused by an overstimulated sympathetic nervous system, excessive catecholamine release, and down-regulation and desensitization of β-adrenergic receptors. Its prognostic significance was highlighted in a study that retrospectively studied patients who had undergone dobutamine stress echocardiogram for pre-OLT work-up. The study revealed that cirrhotic patients who did not reach 82% of their maximum predicted heart rate during dobutamine stress echocardiogram had a higher likelihood of experiencing major adverse cardiac events.^25^

#### Coronary artery disease

Autopsy studies have suggested that cirrhosis might actually offer protection against the development of coronary artery disease (CAD). However, more recent studies using stress imaging, computed tomography coronary angiography, and invasive coronary angiography report a CAD prevalence of up to 29% in cirrhotic patients being assessed for OLT.^26^, ^27^, ^28^ Patients with established CAD undergoing OLT experience higher rates of mortality with 1 study reporting a post-OLT all-cause mortality of 26.7%, compared with 9.6% in patients without CAD.^29^ These findings reveal that patients with cirrhosis are more likely to have subclinical CAD and this highlights the need for rigorous preoperative cardiac risk stratification in this population.

The clinical presentation of ischemic CAD in cirrhotic patients can be challenging to differentiate from symptoms of decompensated cirrhosis. Several recent cohort studies on patients undergoing OLT evaluation revealed a high prevalence of moderate-severe CAD in completely asymptomatic patients.^30^^,^^31^ Thus, recognition of ischemic CAD requires a high index of suspicion and particular attention to CAD risk factors. Traditional cardiovascular risk factors, including, hyperlipidemia, diabetes mellitus, obesity, and tobacco use have become increasingly prevalent among cirrhotic patients. This trend has been attributed to improved survival rates in this population. Moreover, nonalcoholic steatohepatitis cirrhosis, currently the fastest growing indication for OLT, is linked with a higher risk for cardiovascular complications post-OLT.^32^^,^^33^ To further emphasize this, the 2012 American College of Cardiology/American Heart Association guidelines on cardiac evaluation for OLT candidates recommend noninvasive stress testing for this population on the basis of having multiple CAD risk factors, regardless of functional status (Class IIb, Level of Evidence: C).^34^ It should be noted that stress echocardiography yields a low sensitivity and negative predictive value for the identification of obstructive CAD in this population. Interestingly, achieving >85% of the maximal predicted heart rate or a rate pressure product >25,000 during stress does not seem to improve the diagnostic yield of dobutamine stress echocardiogram in patients with advanced cirrhosis.^35^ However, there is evidence that incorporating global longitudinal strain (GLS) and postsystolic shortening during stress, another strain derived parameter, improves sensitivity and overall diagnostic performance.^36^

## Diagnostic approach

The subclinical nature of cirrhosis-induced cardiac dysfunction requires a comprehensive approach incorporating laboratory tests, clinical assessment, electrocardiography, and imaging studies. The clinical syndrome of cardiac dysfunction in cirrhosis, termed cirrhotic cardiomyopathy, was first recognized over 3 decades ago, and its diagnostic criteria have continued to evolve with advancements in imaging. However, a universally accepted definition remains uncertain, with ongoing debate over the specific criteria. Diagnostic criteria aside, cirrhotic cardiomyopathy is largely a syndrome of heart failure with preserved ejection fraction and electrophysiologic abnormalities with many patients experiencing an inability to appropriately augment cardiac function during stress.

### Electrocardiography

As described in detail in the previous text, cardiac electrophysiologic disturbances are prevalent in cirrhotic patients and QTc prolongation is one of the most frequently observed abnormalities. Subsequent interventions often performed in patients with ESLD result in varying effects on the QTc. For example, transjugular intrahepatic portosystemic shunt often prolongs the QTc and OLT results in shortening or normalization of the QTc.^37^^,^^38^ The duration of the QT interval is influenced by heart rate variability, with shorter R-R intervals resulting in a decreased QT interval. QT interval correction is essential to account for heart rate variability and Bazett’s correction formula is most used in practice. However, this formula has been criticized for its undercorrection at slower heart rates and overcorrection at faster heart rates.^39^ The Fridericia formula is an alternative method for correcting the QT interval based on the R-R interval and has demonstrated superior accuracy in patients with cirrhosis.^39^

### Biomarkers

Assessment of biomarkers can provide supporting evidence for the presence of cirrhosis-induced cardiac dysfunction. Natriuretic peptides, including brain natriuretic peptide (BNP) and N-terminal pro-BNP, are produced by cardiomyocytes and released in response to wall strain. Systemically, this results in natriuresis, diuresis, and vasodilation. These values are particularly useful in the diagnosis and management of heart failure and have been linked with increased risk of all-cause mortality.^40^ In the context of cirrhosis, elevated BNP is correlated with the severity of liver disease and found to be associated with increased post-OLT mortality.^41^^,^^42^ Routine monitoring of pre-OLT BNP can provide important prognostic information and may serve as a practical biomarker for mortality risk stratification in OLT.^43^

In addition, troponin elevation serves as an important biomarker to identify cardiomyocyte damage. In cirrhotic patients, Troponin I has been associated with subclinical LV myocardial injury and troponin T has been linked to higher 1-year mortality following an emergency department visit.^44^^,^^45^ In addition, high sensitivity troponin T has emerged as a reliable marker of disease severity in cirrhosis.^46^ Finally, pre-OLT troponin I levels have shown a strong correlation with post-OLT major adverse cardiac events.^47^

### Echocardiography

#### Systolic function

Echocardiography is an essential tool for evaluating cardiac dysfunction in patients with ESLD, particularly in those undergoing evaluation for OLT. Traditionally, LV systolic function is assessed using volume-based LV ejection fraction as the standard measurement. This parameter, however, is limited by interobserver variability and certain pathophysiological states—such as LV hypertrophy with a small LV cavity size—where a normal LV ejection fraction may mask a reduced stroke volume.^48^

Advancements in cardiac imaging, including myocardial strain, have introduced a modern approach for the evaluation of systolic function to overcome these challenges.^49^ The mechanics of myocardial performance involves a complex combination of deformation, or strain, that includes longitudinal, circumferential, and radial shortening to produce ejection. Altogether, this offers a more comprehensive view of systolic function.^48^ Conceptually, myocardial strain is simply a measurement of change in myocardial fiber length through the cardiac cycle.^50^ Myocardial strain is calculated using speckle tracking echocardiography, which tracks the movement of acoustic reflectors, or "speckles," within the myocardial tissue and has emerged as a more advanced technique for calculating myocardial strain.^50^ GLS is the most commonly reported myocardial strain parameter, and reflects an average of the strain values from multiple regions. GLS can detect systolic dysfunction despite normal LV ejection fraction and serves as an early marker for LV contractile abnormalities. This makes it a potentially useful tool in the assessment of systolic dysfunction in cirrhotic patients.^51^ A recent meta-analysis reviewed 19 studies that evaluated GLS in cirrhotic patients and found that this population had a 1.66% lower absolute GLS after sensitivity analysis.^52^

GLS has also been evaluated in relation to cirrhosis severity by Child-Pugh and Model for end-stage liver disease scores and studies have revealed varying results. Some studies found no significant differences in GLS across Child-Pugh classes or model for end-stage liver disease scores. However, other studies identified higher absolute GLS values in patients with more severe liver disease.^53^, ^54^, ^55^, ^56^ Similarly, the prognostic value of GLS in cirrhosis has been studied and also yielded mixed results. Some studies have shown reduced GLS to be an independent predictor of mortality in patients undergoing OLT assessment and transjugular intrahepatic portosystemic shunt placement. However, other studies found no significant association with outcomes. These inconsistencies highlight the need for further research to clarify the relationship between GLS, cirrhosis severity, and prognosis.^56^, ^57^, ^58^

#### Diastolic function

LV diastolic dysfunction is clinically significant because it has been linked to an increased risk of allograft rejection, graft failure, and mortality.^59^ Echocardiographic assessment of LV diastolic dysfunction is critical in the cardiac assessment of patients with cirrhosis because it is often an early indicator of various cardiovascular pathologies. The cirrhotic heart undergoes structural remodeling and histological changes which contribute to the development of LV diastolic dysfunction. This condition is characterized by insufficient LV filling and subsequent reduction of LV stroke volume. Over time, this can progress to LV systolic dysfunction. Many extracardiac factors, including vasodilation/vasoconstriction, changes in preload/afterload, metabolic derangements, and medications are seen in cirrhosis and can influence ventricular filling.

LV diastolic dysfunction can be assessed using several echocardiographic techniques, including early rapid filling (E), late filling from atrial contraction (A), deceleration time, and isovolumetric relaxation time. The rate at which the E-wave velocity decreases is faster when the LV is less compliant.^60^ Tissue Doppler imaging measures myocardial tissue velocity as opposed to blood flow velocities.^61^ Early mitral annulus diastolic velocity (e′) is regarded as the most reliable measurement of LV diastolic dysfunction because it is less influenced by volume changes. Prolonged LV relaxation is linked to a reduced e′ velocity and the resultant E/e′ ratio has proven to be a valuable tool for estimating LV filling pressures.^62^ In the context of cirrhosis, a retrospective study found abnormal septal e′ to be the most predictive echocardiographic parameter for major adverse cardiac events following OLT.^63^ Further, the E/e′ ratio has been shown to be positively correlated with severity of liver disease and incidence of post-OLT atrial fibrillation/flutter.^64^, ^65^, ^66^ It also serves as an independent predictor of all-cause mortality post-OLT.^67^

Increased LV filling pressures are reflected back into the left atrium causing dilation. This enlargement is quantified using the left atrial volume index and a value >34 mL/m^2^ reflects diastolic dysfunction.^68^ Increased left atrial volume index has been linked with a higher risk of heart failure following OLT.^69^ The resultant increased left atrial pressures can be transmitted back into the pulmonary artery and measurement of these pressures can also provide further insight into the extent of diastolic dysfunction. Peak tricuspid regurgitation velocity assessed via continuous wave Doppler, serves as a surrogate for pulmonary artery systolic pressure estimation and a value >2.8 m/s can indicate elevated left atrial pressures. Of note, patients with ESLD often have complex systemic conditions, including portopulmonary hypertension, which can cause elevated right ventricular pressures. Therefore, peak tricuspid regurgitation velocity should be interpreted in the context of other diastolic parameters to ensure accurate assessment.^68^

### Cardiac magnetic resonance imaging

Cardiac magnetic resonance imaging (CMR) has become the leading method for evaluating cardiac function and structure in various types of cardiomyopathies.^70^ Beyond its accuracy in quantifying chamber size and LV ejection fraction, CMR provides advanced myocardial tissue characterization through techniques such as late gadolinium enhancement, extracellular volume fraction quantification using T_1_ mapping, T_2_ mapping, and T_2_∗ mapping.

Late gadolinium enhancement describes areas of the myocardium with high signal intensity appearing approximately 10 minutes following intravenous administration of gadolinium.^71^ This technique can detect areas of fibrosis, scarring, or necrosis and typical patterns for various cardiomyopathies have been described. The distinct patterns of late gadolinium enhancement in patients with cirrhosis-induced cardiomyopathy and their prognostic significance remains poorly understood. One study utilized CMR to assess late gadolinium enhancement in 20 patients with ESLD, revealing a patchy distribution pattern similar what is seen in myocarditis.^71^

Extracellular volume fraction quantification using T_1_ mapping is estimated by measurement of myocardial and blood T_1_ before and after administration of a contrast agent has emerged as a more effective way of detecting fibrosis. It has been shown to be a strong predictor of poor outcomes in several cardiac diseases.^72^ In patients with cirrhosis, myocardial extracellular volume fraction is increased when compared with control subjects, correlated with increasing Child-Pugh class, and is associated with poor transplant-free survival.^73^ Interestingly, extracellular volume fraction normalization was observed 1 year post-OLT, highlighting its dynamic nature.^74^

Finally, CMR is capable of detecting and quantifying iron overload utilizing T_2_∗ relaxation times, with lower values indicating overload.^75^ This is particularly relevant for patients with ESLD caused by hereditary hemochromatosis, which carries poorer post-OLT survival rates compared with those with ESLD from other causes. This increased mortality has been attributed to the systemic effects of iron overload, including heart failure and arrhythmias.^76^ Of note, studies have also shown that significant iron overload can occur in ESLD unrelated to hemochromatosis, making T_2_∗ relaxation times clinically useful for cardiovascular assessment pre-OLT. A study on OLT candidates found that those with model for end-stage liver disease ≥25, Child-Pugh Class C, and an LV ejection fraction <65% had a 5-fold higher likelihood of a T2∗ value below 20 ms.^77^

## Clinical Implications

### Hepatorenal syndrome

Hepatorenal syndrome is a form of kidney dysfunction seen in cirrhotic patients and characterized by a decline in glomerular filtration rate caused by severe renal vasoconstriction in the absence of structural kidney damage. Cirrhosis induced cardiac dysfunction has been implicated in the development of hepatorenal syndrome when precipitated by spontaneous bacterial peritonitis. A prospective study of 23 patients with spontaneous bacterial peritonitis found that those who developed renal failure had lower cardiac output, which continued to decline postspontaneous bacterial peritonitis resolution. These findings suggest that circulatory dysfunction and renal failure in spontaneous bacterial peritonitis are primarily driven by reduced cardiac output.^78^ A follow-up study by the same group monitored 66 cirrhotic patients with ascites and found that those who developed hepatorenal syndrome had significantly lower cardiac output, which emerged as an independent predictor of hepatorenal syndrome progression.^79^ Finally, a later study found that reduced cardiac index was associated with poor outcomes and renal failure in patients with advanced cirrhosis.^80^ The decline in cardiac output leads to reduced renal perfusion, a condition worsened by compensatory mechanisms that enhance contractility through the sympathetic nervous system and drive renal sodium and water retention via the renin-angiotensin-aldosterone system.

### Transjugular intrahepatic portosystemic shunt

Transjugular intrahepatic portosystemic shunt placement in patients with ESLD introduces a sudden increase in preload, which results in significant hemodynamic changes. By shunting blood from the congested splanchnic circulation directly into the systemic circulation, transjugular intrahepatic portosystemic shunt exacerbates the pre-existing hyperdynamic state by increasing preload, heart rate, and cardiac output postprocedure.^81^ Although transjugular intrahepatic portosystemic shunt reduces portal hypertension and its complications, the abrupt hemodynamic shift can overwhelm the heart and impede its ability to accommodate the increased preload which can precipitate cardiac dysfunction. A study found that cirrhotic patients with refractory ascites who underwent transjugular intrahepatic portosystemic shunt had an increased risk of heart failure compared with those treated with repeated paracentesis and albumin.^82^ Conversely, another study using CMR showed that while increased preload from transjugular intrahepatic portosystemic shunt led to cardiac chamber enlargement, these changes did not result in cardiac impairment at follow-up.^83^

### Orthotopic liver transplantation

Although OLT is the definitive treatment for ESLD, the procedure itself imposes substantial cardiovascular stress caused by surgical clamping, hemorrhage, volume resuscitation, and the hemodynamic shifts associated with ischemia and reperfusion. The restoration of liver function and normalization of portal pressure with OLT leads to a significant rise in blood pressure and peripheral vascular resistance, resulting in increased preload and afterload. These rapid hemodynamic shifts put OLT recipients at an increased risk of perioperative complications, including acute heart failure, myocardial infarction, and life-threatening arrhythmias.^12^ However, in the long term, OLT has been shown to result in cardiovascular recovery with improvements in systolic and diastolic dysfunction as well as QT prolongation within 3 to 6 months of surgery.^84^ Effective cardiovascular management before, during, and after OLT is essential for achieving favorable outcomes. Several studies have investigated clinical and diagnostic predictors of outcomes and, while each has its limitations, they consistently indicate enhanced cardiovascular function following OLT.

### Portopulmonary hypertension and hepatopulmonary syndrome

Pulmonary complications of cirrhosis include portopulmonary hypertension and hepatopulmonary syndrome. Portopulmonary hypertension occurs in the setting of portal hypertension and is defined by an elevated mean pulmonary arterial pressure and increased pulmonary vascular resistance, while hepatopulmonary syndrome is characterized by intrapulmonary vascular dilations and arteriovenous shunting that lead to hypoxemia. To start, portopulmonary hypertension is an uncommon complication of ESLD and is associated with significant morbidity and mortality, primarily caused by right ventricular failure.^85^ The proposed mechanism for its development associates elevated cardiac output with increased shear stress on pulmonary vasculature which stimulates the release of vasoactive mediators.^86^ The right ventricle is a thin walled structure that is highly sensitive to the changes in afterload seen in portopulmonary hypertension, which can lead to RV dilation, increased central venous pressure, and congestive hepatopathy. In patients with severe portopulmonary hypertension, optimizing mean pulmonary artery pressures to <35 mm Hg with diuretic agents and pulmonary vasodilators is recommended before OLT, which is typically curative for both portopulmonary hypertension and hepatopulmonary syndrome. Studies evaluating the impact of hepatopulmonary syndrome with cardiac function have linked right ventricle diastolic dysfunction and increased left atrial volume to the presence of hepatopulmonary syndrome.^87^

## Management

Currently, OLT is the sole definitive intervention for the management of cirrhosis-induced cardiac dysfunction because no widely accepted treatment strategies or standardized protocols have been established for its management. Supportive care and symptom management with supplemental oxygen, salt restriction, and diuretic therapy is the mainstay of treatment.^88^ Although the use of guideline-directed medical therapy is the cornerstone for management of noncirrhotic heart failure, its application in patients with cirrhosis is limited by contraindications and safety concerns. For example, the American Association of the Study of Liver Diseases guidelines advise against use of angiotensin-converting enzyme inhibitors and angiotensin receptor blockers in patients with ascites caused by the risk of further reducing effective arterial volume and renal perfusion, which can precipitate acute kidney injury and worsen survival outcomes.^89^

β-blockers are commonly used for the prevention of variceal bleeding in cirrhotic patients. However, their use requires careful consideration in the setting of decompensated cirrhosis, particularly in the presence of refractory ascites, spontaneous bacterial peritonitis, or severe hypotension caused by their potential adverse effects.^90^ A prospective, randomized trial of 78 patients with an impaired cardiac output response during dobutamine stress echocardiogram found that β-blocker therapy did not improve cardiac function and morphology in patients with cirrhosis-induced cardiac dysfunction.^91^ Moreover, a separate study found nonselective β-blocker use in patients with ESLD and refractory ascites to be linked with a higher risk of mortality.^90^ On the other hand, β-blocker therapy has been demonstrated to reduce QT interval prolongation in cirrhosis and potentially lower the risk of ventricular arrhythmias. However, no studies have established a connection between this effect and improved clinical outcomes.

Aldosterone antagonists are also commonly used in patients with cirrhosis to manage ascites and edema. These agents have demonstrated some benefit in cirrhosis-induced cardiac dysfunction. Specifically, they improve hepatic hemodynamics with a reduction in hepatic venous pressure gradient. Additionally, the positively impact myocardial structure and function, as evidenced by reduced LV wall thickness and LV end-diastolic diameter.^92^

## Unmet Needs and Future Directions

The lack of effective pharmacologic interventions and reliable biomarkers for early detection of cirrhosis-induced cardiac dysfunction can largely be explained by gaps in our current understanding of the distinct pathophysiology behind this disease. In addition, this entity diverges substantially from traditional forms of heart failure and, as described previously, conventional heart failure therapies have shown limited success. This highlights the need for further research tailored specifically to this population.

The latent nature and subsequent stressor-induced clinical manifestation of this condition suggests a maladaptive remodeling process that initially preserves function but later becomes unstable. Research aimed at optimizing cardiac energy metabolism, ATP generation, and substrate flexibility may offer novel therapeutics to enhance cardiac resilience in cirrhosis.^93^

Additionally, emerging data highlight the central role of fibrogenesis in the development of this condition and may serve as a promising therapeutic target.^93^ Recent studies utilizing advanced cardiac and hepatic imaging have revealed that myocardial fibrosis closely mirrors liver fibrosis in cirrhosis, with strong correlations to systemic markers of collagen turnover and inflammation.^94^

Furthermore, oxidative stress, inflammation, and apoptosis have been identified as key mediators of myocardial injury in cirrhosis, highlighting potential avenues for discovery of novel biomarkers and therapeutic targets. Studies have linked the development of cirrhosis-induced cardiac dysfunction with the activation of nuclear factor-κB and subsequent release of proinflammatory cytokines, including TNF-α and IL-1β, which up-regulate inducible nitric oxide synthase and subsequent nitric oxide release.^95^ As in the previous text, cirrhotic patients exhibit chronically elevated nitric oxide levels, and excess nitric oxide has been linked with promoting inotropic and chronotropic incompetence.^95^ Measuring serum biomarkers associated with these pathways may aid in early detection of cirrhosis-induced cardiac dysfunction by reflecting underlying inflammatory and oxidative activity, though more research is needed. Galectin-3, a β-galactoside-binding lectin, has emerged as a novel biomarker in this condition. Growing evidence suggests an association with Galectin-3 and the pathogenesis of both systolic and diastolic function.^96^^,^^97^ Further, serum levels of Galectin-3 have been found to be significantly increased in patients with cirrhosis. Functionally, Galectin-3 serves as a proinflammatory mediator, stimulating the production of TNF-α.^98^ Additionally, Galectin-3 has been shown to up-regulate the expression of fibrillar collagen, which as described in the previous text, is closely linked to diastolic dysfunction.^98^ Further research is needed in identifying biomarkers associated with cirrhosis-induced cardiac dysfunction, which offer diagnostic and/or prognostic value. With respect to therapeutic options, preclinical studies utilizing agents targeting these pathways, including antioxidants, anti-inflammatory, and antiapoptotic agents have shown promise and warrant further investigation.^99^

## Conclusions

Cirrhosis-induced cardiac dysfunction is an under-recognized entity and characterized by impaired contractility, diastolic dysfunction, and electrophysiological disturbances. This review has examined the intricate relationship between the liver and the heart, with a particular focus on the impact of the former on the latter. We have explored the role of portal hypertension, systemic inflammation, and hemodynamic alterations in driving cardiovascular abnormalities. The pathophysiology involves molecular and structural changes, which include β-adrenergic receptor down-regulation, myocardial fibrosis, and altered ion channel function. Collectively, these alterations ultimately impair cardiac performance.

Diagnostic challenges exist because the cardiac dysfunction seen in this population is often subclinical. However, recent advances in cardiac imaging modalities and the utilization of appropriate biomarkers have enhanced our ability to detect early cardiac dysfunction. From a clinical standpoint, cardiac dysfunction in the setting of cirrhosis significantly impacts outcomes in procedures such as OLT and transjugular intrahepatic portosystemic shunt. This underscores the need for rigorous preoperative cardiovascular assessment.

Although standardized treatment guidelines are lacking, OLT remains the definitive intervention and frequently leads to cardiovascular recovery. Future research should focus on better understanding the molecular mechanisms behind this syndrome, refining diagnostic criteria, and developing targeted treatments to improve patient outcomes. Effective management requires close collaboration between cardiologists, hepatologists, and transplant surgeons to optimize care and improve patient outcomes. Clinicians must look beyond the liver and recognize that a cirrhotic patient’s heart is just as vulnerable. In this intricate balance between 2 vital organs, awareness and early intervention may be our best lifelines.

## Funding support and author disclosures

The authors have reported that they have no relationships relevant to the contents of this paper to disclose.
